# Intimate partner violence against women during and after pregnancy: a cross-sectional study in Mumbai slums

**DOI:** 10.1186/1471-2458-13-817

**Published:** 2013-09-09

**Authors:** Sushmita Das, Ujwala Bapat, Neena Shah More, Glyn Alcock, Wasundhara Joshi, Shanti Pantvaidya, David Osrin

**Affiliations:** 1 Society for Nutrition, Education and Health Action (SNEHA), Urban Health Centre, 60 Feet Road, Dharavi, Mumbai 400017, Maharashtra, India; 2 Institute for Global Health, UCL Institute of Child Health, 30 Guilford Street, London WC1N 1EH, UK

**Keywords:** Intimate partner violence, Domestic violence, India, Mumbai, Maternal health, Slums

## Abstract

**Background:**

At least one-third of women in India experience intimate partner violence (IPV) at some point in adulthood. Our objectives were to describe the prevalence of IPV during pregnancy and after delivery in an urban slum setting, to review its social determinants, and to explore its effects on maternal and newborn health.

**Methods:**

We did a cross-sectional study nested within the data collection system for a concurrent trial. Through urban community surveillance, we identified births in 48 slum areas and interviewed mothers ~6 weeks later. After collecting information on demographic characteristics, socioeconomic indicators, and maternal and newborn care, we asked their opinions on the justifiability of IPV and on their experience of it in the last 12 months.

**Results:**

Of 2139 respondents, 35% (748) said that violence was justifiable if a woman disrespected her in-laws or argued with her husband, failed to provide good food, housework and childcare, or went out without permission. 318 (15%, 95% CI 13, 16%) reported IPV in the year that included pregnancy and the postpartum period. Physical IPV was reported by 247 (12%, 95% CI 10, 13%), sexual IPV by 35 (2%, 95% CI 1, 2%), and emotional IPV by 167 (8%, 95% CI 7, 9). 219 (69%) women said that the likelihood of IPV was either unaffected by or increased during maternity. IPV was more likely to be reported by women from poorer families and when husbands used alcohol. Although 18% of women who had suffered physical IPV sought clinical care for their injuries, seeking help from organizations outside the family to address IPV itself was rare. Women who reported IPV were more likely to have reported illness during pregnancy and use of modern methods of family planning. They were more than twice as likely to say that there were situations in which violence was justifiable (odds ratio 2.6, 95% CI 1.7, 3.4).

**Conclusions:**

One in seven women suffered IPV during or shortly after pregnancy. The elements of the violent milieu are mutually reinforcing and need to be taken into account collectively in responding to both individual cases and framing public health initiatives.

## Background

A violation of human rights with health and development impacts [1], violence against women is a global public health concern [2]. Intimate partner violence (IPV), defined as “behaviour within an intimate relationship that causes physical, sexual or psychological harm, including acts of physical aggression, sexual coercion, psychological abuse and controlling behaviours” [3,4], is suffered by between 15% and 71% of women at some point in their lives [3].

### IPV in India

Although population-based surveys underline the ubiquity of IPV, its occurrence and impacts are frequently hidden and the figures are usually underestimates [5]. India’s third National Family Health Survey (NFHS-3 2005–2006) suggested that 33% of ever-married women aged 15–49 years had faced IPV at some point over the age of 15 [6]. A second large cross-sectional survey involving over 14 000 women in 18 states yielded an estimate of 39% [7]. Other community-based surveys suggest a prevalence of physical IPV of 35-60% [8-10].

### IPV in urban India

A secondary analysis of NFHS-3 data suggested that 28% of ever-married urban women aged 15–49 years had experienced physical violence in the last year [11]. There is less information on IPV in the slums that characterize India’s cities. The NFHS-3 estimated the lifetime prevalence against ever-married women at 23% in Mumbai’s slum population [6]. The figure was 54% in a Kolkata slum [12]. Over shorter recall periods, 37% of women in a Mumbai slum survey reported violence in the preceding year (verbal 32%, physical 23%, and sexual 9%) [13], compared with 62% in Pune [14], 17% in Kolkata [15], and 27% in Bangalore in the preceding six months [16].

### IPV during maternity

Pregnancy does not protect women from IPV [17-19]. A global systematic review described a prevalence during pregnancy of 1-20% [20], a review of studies in Asian countries 4-48% [21], and a review of Indian studies 21-28% [22].

### Consequences of IPV during maternity

IPV leads to both acute injuries and profound longer-term challenges to health and wellbeing [23,24]. Studies from India suggest that women who experience physical IPV during pregnancy are more likely to suffer depression and mental ill-health [25]. They are more likely to miscarry, have premature labor [26], and deliver low birth weight infants [27,28]. The survival of their infants is reduced in the perinatal [29,30], neonatal [29,31], infant [32], and childhood periods [33]. Women who report IPV are also less likely to have prenatal care [34,35], may be more likely to terminate pregnancy [36], and are at greater risk of sexually transmitted infection [37,38].

### Risk factors for IPV

**Figure 1:**
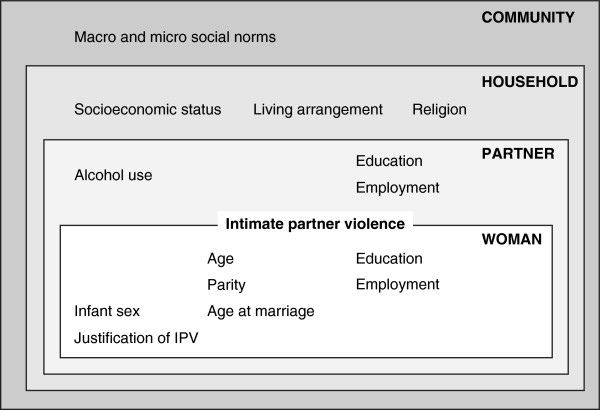
Conceptual framework for determinants of intimate partner violence included in the dataset.

Figure 1 categorizes factors available in our dataset that have been described as potential determinants of IPV in India and included in recent reviews [35,39]. Each level interacts with others. For example, improvements in women’s education may lead to changes in societal attitudes to IPV, and have a bearing on employment and socioeconomic status. At community level, IPV appears to be less common in urban than in rural environments [11,40]. Although it is difficult to quantify, several studies have suggested that an absence of social support might make women more vulnerable to IPV [10,41,42].

At the household level, there is a consensus that poverty increases the risk of IPV. This is seen in Indian studies of IPV in general [10,43-48], in pregnancy [7,11], and in slum communities [12,15]. IPV has been described as more likely to be experienced by women from lower castes [43], larger families, and certain faith groups [7,11,18,49]. There is some evidence that women who have witnessed abuse in their families are more likely to experience it themselves [10,45].

Having more educated partners appears to protect women against IPV [42,50]. The same is true of employment, in that women whose partners are in work are less likely to suffer IPV. Spousal alcohol or drug use is a risk factor in many studies [10,41-43,47,51,52], including those that consider IPV in pregnancy [7,11,13], and in slum communities [12,15]. Risk-taking behavior such as spousal gambling [7] and extramarital sex increase the risk of IPV [15,53].

Women who marry younger (under 18 years in most studies) are at greater risk of IPV [11,54-56], although this finding is not always replicated [57] and older women are also at increased risk [13,46,47]. Longer marriages may be associated with increased [15] or decreased risk [7]. As with their partners, women with less education are at greater risk of IPV [7,11,13,18,27,45-47,50,54]. It has been suggested that women who are more educated than their husbands are at greater risk [50], but this has not been substantiated and a recent study found the opposite [58]. The effects of other risk factors are unclear, particular examples in the Indian context being son preference and women’s employment. We discuss these later.

SNEHA (Society for Nutrition, Education and Health Action) is a non-government organization working to improve the health of women and children in Mumbai’s disadvantaged settlements, in four domains: prevention of violence against women and children, reproductive health, maternal and newborn health, and childhood nutrition. We offer a range of counseling, legal, and liaison services for women and children experiencing violence. Despite our work in the area for over a decade, we had little information on the prevalence of IPV within slum communities. We had also become concerned about the impact of IPV on women’s experience of maternity. During a community-based program that focused on maternal and newborn health [59], we interviewed women after childbirth. We included a series of questions about violence within a larger questionnaire. Our objectives were to describe the prevalence of IPV during pregnancy and postpartum in an urban slum setting, to review potential risk factors in the urban context, and to explore its effects on maternal and newborn health.

## Methods

### Setting

The capital of Maharashtra state, Mumbai has 12.4 million residents. Although 63% of households fall into India’s highest wealth quintile [6], 53% of residents live in slums [60]. Indicators of women’s status such as literacy, workforce participation, age at marriage, and healthcare seeking tend to be better than the national averages. We used data collected during a cluster randomized controlled trial of community mobilization to improve maternal and newborn health [59,61]. The trial involved 48 slum clusters, each of ~1200 households, covering a population of ~283 000. 26% of homes were of insubstantial fabric (corrugated iron, planking, tarpaulin) and sizeable proportions did not have metered electricity (28%), access to individual or communal piped water (21%), or individual toilet facilities (94%). More than one-third of clusters were adjacent to hazards such as railway lines, garbage dumps and polluted bodies of water.

### Data collection

A registration system monitored live births, stillbirths, maternal and neonatal deaths in all 48 slum clusters. Births were identified by 99 locally resident women, covering ~600 households each. After confirming a birth, one of 12 researchers visited mothers 6 weeks later for a postnatal interview. The interview included questions on demographic characteristics, socioeconomic indicators, maternal and newborn care, and was framed as a women’s health study with a particular focus on maternal and newborn health. After an explanation of the data collection activities, participants were asked for verbal consent to interview and were assured of data confidentiality. Completed interviews were checked by supervisors and project officers, both systematically and through random visits, and were entered in a database in Microsoft Access (Microsoft Corporation). Information provided by the participants remained confidential and no analyses or outputs included their names. Access to data was restricted to the analysts and was password protected.

Because information on the epidemiology of IPV is limited, particularly at disaggregated urban levels, we added a module to the routine questionnaire. All women who gave birth from March to September 2009 and consented to interview received the module, which included questions about justifications for IPV, instances of physical, emotional, or sexual IPV in the preceding year, injuries, help seeking, and spousal drug and alcohol use. The questions were adapted from existing versions in the Demographic and Health Surveys [6].

We followed World Health Organization ethical and safety recommendations [62]. Participants were told that the study was about maternal and newborn health, and that women in the study area who had given birth in a certain period would be approached for interview. During the interview, participants were informed of the nature and potential sensitivity of the IPV module and consent was taken before administering it. One woman per household received the module. Most of the interviews were conducted in participants’ homes. The researchers made efforts to interview women alone, but the density of slum homes and a desire to make respondents comfortable meant that there were limits to their ability to achieve privacy. At the first visit, if family members were present and the researcher was concerned about privacy, she asked the respondent for a suitable time to make a second visit. If family members were present again, information about the maternal and newborn health study was shared with them. The researcher told them that some questions might be embarrassing for the participant to answer in the presence of others. The interview began with the routine questionnaire and, when family members were reluctant to leave, the researcher encouraged them to stay for a while to reassure themselves about the nature of the questions. Most family members were generally amenable to leaving before the IPV module. The researchers carried a resource list of support services near the study area. They visited providers and informed them about the study, and that their help might be needed. All participants received information on resources, irrespective of whether or not they reported violence. They were told that the information could help them to support other women if they suffered violence, and referral was arranged if requested.

Given available estimates of the prevalence of IPV, a sample size of 1800 would give an estimated proportion with a precision of 5% at a confidence level of 95%. Recruitment was planned to be sequential until 300 questionnaires had been collected in each of the six municipal wards across which the 48 clusters were distributed. We assumed a high attrition rate because of the sensitive nature of the questions, and interviewed more women than the required sample size.

### Variables

We defined physical IPV according to the WHO Multi-Country Study [63], as an instance in which her partner beat, punched, kicked, dragged, or slapped a woman, twisted her arm, pulled her hair, hit her with an object, choked, burned, or physically restrained her. Emotional IPV was defined as an instance in which a spouse showed jealousy, humiliated his partner in front of others, accused her of infidelity, threatened to evict her or actually did so, threatened her or her children with violence, or forcibly took something from her. Sexual IPV was defined as an instance in which a woman was forced to have sex with her partner when she did not want to. Respondents were asked the questions about IPV in the preceding year. The timing of the interview meant that the preceding year encompassed less than two months before the pregnancy, pregnancy itself, and the postpartum period. For each type of IPV, a subsidiary question on frequency followed (*once, sometimes, often*). We also asked respondents who reported physical IPV whether it had happened more before, during or after pregnancy. We asked about injuries or complications that might have resulted from it (cuts, bruises, pain, burns, wounds, fractures and dental trauma, fetal death, premature labour, vaginal bleeding), and whether it led to problems (self-care, infant care) such as prevention of breastfeeding. We asked whether respondents had sought help from family members, friends or neighbours, local or religious bodies, women’s or other non-government organizations, doctors, the police, lawyers or a helpline.

Socioeconomic status was described by asset scores assigned to respondents on the basis of standardized weights for the first component of a principal components analysis, ordered and divided into quintiles [64,65]. Assets included a range of consumer durables and house ownership and construction. The remaining variables included miscarriage (cessation of pregnancy before 22 completed weeks) in a previous pregnancy, any reported illness during the index pregnancy, receipt of any prenatal care, home delivery, preterm delivery before 37 completed weeks of gestation, low birth weight (< 2500 g), infant sex, and use of a modern family planning method in the three years preceding the index pregnancy (oral contraceptive pill, condoms, intrauterine device, injectable or implantable contraception).

### Statistical analysis

We summarized responses to questions about IPV with frequencies and percentages, categorized according to type of IPV. Analysis of determinants followed the conceptual framework in Figure 1. We hypothesized that the odds of IPV during maternity would decrease with rising socioeconomic status, woman’s age, age at marriage, parity, and education, and with her husband’s education and employment; would increase if her husband used alcohol or drugs; and would be affected in an unspecified direction by family structure and religion. We entered IPV as a binary dependent variable in multivariable logistic regression models with a random effect for cluster. Hypothetical determinants were entered as independent variables in both univariable and multivariable models [66]. At *household* level, socioeconomic quintile, family unit, and religion were entered as categorical indicator variables. At *woman* level, age-group, schooling, age-group at marriage, and parity were entered as categorical indicator variables, and employment as a binary variable. At *partner* level, husband’s schooling was entered as a categorical indicator variable, and employment and alcohol use as binary variables. We did not expect the intervention under test in the trial in which the study was nested to affect IPV. Including a covariate for allocation in the analysis made no substantial difference to the findings, and it has not been included in the analyses presented.

In an additional analysis, we hypothesized that having a female baby might increase the odds of IPV, during pregnancy if the infant sex was known, or postpartum. We therefore added an independent variable for infant sex to a model including the covariates already mentioned. We also hypothesized that IPV might be more likely to occur in an environment in which a woman had had a previous miscarriage. Again, we added an independent miscarriage variable to the existing model.

Exploratory analyses examined whether IPV was a potential determinant of a series of outcomes. First, we hypothesized that IPV would increase the odds of illness during the index pregnancy, preterm birth, low birth weight and home delivery, and that it would reduce the odds of prenatal care. Second, we hypothesized that women who reported IPV would be more likely to justify it. Third, we hypothesized that women who reported IPV would be less likely to have used modern methods of family planning in the preceding three years. We entered each of these as a binary dependent variable, IPV as an independent variable, and included covariates shown to be associated (at p < 0.1) in the analysis summarized in Table 1: socioeconomic quintile, religion, maternal age (forced into the model in view of its intuitive importance) and employment, and husband’s alcohol use. Analyses were done in Stata 12 (College Station, TX) and presented as odds ratios with 95% confidence intervals.

**Table 1:** Opinions on the justifiability of wife beating, based on responses from 2139 slum-dwelling women interviewed at around 6 weeks postpartum

**A husband is justified in hitting or beating his wife if…**	**n (%)**
She shows disrespect for her in-laws	671 (31)
She doesn’t cook food properly	398 (19)
She goes out without telling him	413 (19)
She argues with him	391 (18)
She does not fulfill his expectations of her household or childcare duties	347 (16)
He loves her, so he has a right to beat her	291 (14)
She refuses to have sex with him	165 (8)
He suspects her of being unfaithful	146 (7)
He is drunk	136 (6)
He or his family are unhappy with her marriage contribution	102 (5)
**Respondents**	2139 (100)

Individual proportions do not sum to 100% because respondents could respond positively to more than one question.

### Ethical approval

Data for the study originated from a larger process of data collection approved by the Municipal Corporation of Greater Mumbai and the Independent Ethics Committee for Research on Human Subjects (Mumbai committee, reference IEC/06/31).

## Results

Data were provided by 2591 women over 9 months in 2009. Interviews were not achieved in 452 cases (17%), in which women consented to and completed the questionnaire on maternal and newborn health, but withdrew from the IPV module either before or shortly after it began. Lack of privacy played a part in this (310, 12%). There were no appreciable differences in socioeconomic status, education, or age between respondents and non-respondents. The analysis was based on information provided by 2139 women.

Table 1 summarizes responses to the question, “Sometimes a husband is annoyed or angered by things that his wife does. In your opinion, is a husband justified in hitting or beating his wife in the following situations?” Over one-third of respondents felt that IPV was justifiable in some situation (768, 36%), including 748 (35%) who said that it would be justified if a woman disrespected her in-laws or argued with her husband, failed to provide good food, housework and childcare, or went out without his knowledge. Overall, 318 (15%, 95% CI 13, 16%) women reported facing IPV in the year that included pregnancy and the postpartum period (Table 2). Physical IPV was reported by 247 (12%, 95% CI 10, 13%), and coercive sex by 35 (2%, 95% CI 1, 2%). Emotional violence was reported by 167 women (8%, 95% CI 7, 9). Slaps, kicks and punches were the most common forms of physical violence.

**Table 2:** Intimate partner violence in the preceding year reported by 2139 slum dwelling women interviewed at around 6 weeks postpartum

	**n (%)**
**Physical, emotional or sexual intimate partner violence**	**318 (15)**
**Physical IPV**	**247 (12)**
Slapped	212 (10)
Beaten	97 (5)
Punched	75 (4)
Kicked	80 (4)
Hair pulled	53 (2)
Dragged	35 (2)
Arm twisted	42 (2)
Threatened or attacked with a household object or knife	17 (<1)
Choked	11 (<1)
Burned	2 (<1)
Locked up or tied up	6 (<1)
**Emotional intimate partner violence**	**167 (8)**
Jealousy or anger if she talked to other men	120 (6)
Humiliated in front of others	62 (3)
Insulted and made to feel bad about herself	44 (2)
Accused of being unfaithful	37 (2)
Asked to leave the home	36 (2)
Thrown out of the home	24 (1)
Threatened with harm to herself or someone close to her	24 (1)
Threatened with harm to or denial of access to her children	23 (1)
Forcible removal of possessions	21 (1)
**Sexual intimate partner violence**	**35 (2)**
Forced to have sex	35 (2)
**Respondents**	2139 (100)

Individual proportions do not sum to 100% because respondents could have experienced more than one form of violence.

Table 3 shows that about one-third of women said that IPV reduced during pregnancy and the postpartum period, but that 69% said that it either remained at the same level or increased. When asked if IPV was more likely when spouses had been drinking alcohol, 58/149 (39%) women said that it was. Help-seeking to stop IPV was limited (15%) and mostly within the natal family (13%). No women had sought help from a friend or neighbour, religious leader, doctor, lawyer or helpline; 5 had involved the police. Half of women who reported physical IPV in the last year said that it happened sometimes or often. The commonest physical sequelae were pain, cuts and bruises, and a few women reported complications such as vaginal bleeding or early labour. Medical treatment was needed by about one-fifth of women who reported physical IPV. Some said that their ability to care for themselves (17%) or their baby (8%) had been compromised.

**Table 3:** Timing, frequency, and help seeking for intimate partner violence in the preceding year reported by 318 slum-dwelling women, and perceived consequences reported by 247 who reported physical violence, interviewed at around 6 weeks postpartum

	**n (%)**
**Physical, emotional, or sexual intimate partner violence**	**318 (100)**
Relationship of IPV with maternity	
No difference with respect to maternity	147 (46)
More before pregnancy	99 (31)
More during pregnancy	52 (17)
More after delivery	20 (6)
Intimate partner violence more likely when spouse had taken alcohol	58 (18)
Sought help to prevent repeat violence (more than one option possible)	49 (15)
Maternal family	41 (13)
Affinal family	4 (1)
Friend or neighbour	0 (0)
Women’s organization or group, social service organization, local body	6 (2)
Police	5 (2)
Religious leader	0 (0)
Doctor	0 (0)
Lawyer	0 (0)
Helpline	0 (0)
**Physical intimate partner violence**	**247 (100)**
Frequency of physical intimate partner violence in last year	
Once	123 (50)
Sometimes	84 (34)
Often	40 (16)
Sequelae of physical intimate partner violence in last year	79 (32)
Aches and pains	58 (23)
Severe pain	23 (9)
Bruises	20 (8)
Cuts or wounds	18 (7)
Burns	1 (<1)
Fractures or broken teeth	0 (0)
Vaginal bleeding	4 (2)
Early labour	3 (1)
Baby died in womb	2 (<1)
Miscarriage	0 (0)
Affected woman’s ability to care for herself	43 (17)
Affected woman’s ability to care for her baby	19 (8)
Prevented woman from breastfeeding her baby	3 (1)
Sought medical treatment for injury	44 (18)

Table 4 compares the profiles of women who reported emotional, physical or sexual IPV with those who did not. In multivariable models, the odds of IPV were greater for women living in poorer families, the prevalence reaching 28% in the poorest quintile. Greater odds were seen in Muslim families, women in paid employment, and women whose husbands used alcohol. There was no evidence that having a female baby affected the risk of IPV (aOR 1.07; 95% CI 0.82, 1.39). However, in both univariable and multivariable models, women who reported a previous miscarriage were more likely to have reported IPV in the recent pregnancy (aOR 1.76; 95% CI 1.20, 2.58).

**Table 4:** Characteristics of 2139 slum-dwelling women, interviewed at 6 weeks postpartum, who did or did not report physical, emotional or sexual intimate partner violence (IPV) in the preceding year

**Dependent variable**	**Did not report IPV (%)**	**Reported IPV (%)**	**OR**	**(95% CI)**	**Adjusted OR**	**(95% CI)**
**Socioeconomic quintile**						
1 Poorest	343 (19)	90 (28)	1 ref		1 ref	
2	347 (19)	76 (24)	0.832	(0.59, 1.18)	0.934	(0.63, 1.37)
3	376 (21)	53 (17)	0.543	(0.37, 0.80)	0.618	(0.40, 0.95)
4	384 (21)	64 (17)	0.517	(0.35, 0.76)	0.639	(0.41, 1.00)
5 Least poor	371 (20)	44 (14)	0.425	(0.28, 0.64)	0.536	(0.33, 0.88)
*Total*	1821 (100)	318 (100)				
**Family unit**						
Joint	999 (55)	157 (50)	1 ref		1 ref	
Nuclear	819 (45)	160 (50)	1.239	(0.97, 1.59)	1.021	(0.76, 1.37)
*Total*	1818 (100)	317 (100)				
**Religion**						
Hindu	878 (48)	142 (45)	1 ref		1 ref	
Muslim	826 (45)	157 (49)	1.144	(0.85, 1.54)	1.700	(1.22, 2.37)
Other	117 (6)	19 (6)	1.121	(0.64, 1.95)	1.600	(0.89, 2.86)
*Total*	1821 (100)	318 (100)				
**Woman’s age**						
<20 y	117 (6)	23 (7)	1 ref		1 ref	
20-24 y	887 (49)	157 (49)	0.952	(0.58, 1.56)	1.070	(0.61, 1.86)
25-29 y	575 (32)	98 (31)	0.920	(0.55, 1.54)	0.928	(0.50, 1.72)
>29 y	242 (13)	40 (13)	0.896	(0.50, 1.60)	0.746	(0.36, 1.53)
*Total*	1821 (100)	318 (100)				
**Woman’s schooling**						
No schooling	365 (20)	78 (25)	1 ref		1 ref	
<5 y (primary)	82 (4)	31 (10)	1.755	(1.07, 2.88)	1.563	(0.90, 2.71)
5-9 y (secondary)	831 (46)	140 (44)	0.782	(0.57, 1.07)	0.923	(0.65, 1.32)
10 or more y	543 (30)	69 (22)	0.625	(0.43, 0.90)	0.914	(0.59, 1.42)
*Total*	1821 (100)	318 (100)				
**Woman’s age at marriage**						
<20 y	1094 (60)	216 (65)	1 ref		1 ref	
20-24 y	625 (34)	89 (28)	0.730	(0.56, 0.96)	0.858	(0.62, 1.18)
>24 y	102 (6)	13 (4)	0.723	(0.39, 1.33)	0.824	(0.41, 1.68)
*Total*	1821 (100)	318 (100)				
**Woman’s employment**						
Unemployed	1604 (88)	244 (77)	1 ref		1 ref	
Employed	217 (12)	74 (23)	2.320	(1.70, 3.17)	2.008	(1.42, 2.83)
*Total*	1821 (100)	318 (100)				
**Parity**						
1 or 2	1175 (65)	184 (58)	1 ref		1 ref	
3 or more	646 (35)	134 (42)	1.263	(0.98, 1.62)	1.157	(0.82, 1.62)
*Total*	1821 (100)	318 (100)				
**Husband’s schooling**						
No schooling	212 (12)	49 (16)	1 ref		1 ref	
<5 y (primary)	58 (3)	18 (6)	1.298	(0.69, 2.43)	1.375	(0.70, 2.68)
5-9 y (secondary)	676 (38)	128 (41)	0.838	(0.58, 1.22)	1.067	(0.71, 1.60)
10 or more y	833 (47)	114 (37)	0.619	(0.42, 0.91)	1.004	(0.64, 1.59)
*Total*	1779 (100)	309 (100)				
**Husband’s employment**						
Unemployed	18 (1)	7 (2)	1 ref		1 ref	
Employed	1803 (99)	311 (98)	0.570	(0.23, 1.43)	1.001	(0.37, 2.73)
*Total*	1821 (100)	318 (100)				
**Husband drinks alcohol**						
No	1524 (84)	168 (53)	1 ref		1 ref	
Yes	297 (16)	149 (47)	4.884	(3.71, 6.43)	5.223	(3.88, 7.03)
*Total*	1821 (100)	317 (100)				
**Infant sex**						
Male	951 (52)	162 (51)			1 ref	
Female	861 (48)	153 (49)	1.078	(0.84, 1.38)	1.069	(0.82, 1.39)
*Total*	1812 (100)	315 (100)				
**Previous miscarriage**						
No	1620 (89)	261 (82)				
Yes	201 (11)	57 (18)	1.666	(1.20, 2.32)	1.759	(1.20, 2.58)
*Total*	1821 (100)	318 (100)				

OR odds ratio. Ref reference category. ORs and 95% confidence intervals (CI) from logistic regression models with random effect for cluster. Multivariable models included covariates describing socioeconomic status, family unit, religion, woman’s age, schooling, age at marriage, employment, parity, husband’s schooling, employment and alcohol use.

Table 5 summarizes the findings on IPV as a potential determinant of health problems related to pregnancy. Women who reported IPV in the study were more likely to have reported illness during the index pregnancy and use of modern methods of family planning in the preceding 3 years. We speculated that IPV might be associated with parous women having not yet provided a son, or with a substantial age difference or difference in education between wife and husband [15]. We tested several scenarios and found no such associations (data not shown). Women who reported IPV were more than twice as likely to have said that it was justifiable in at least one scenario in Table 1.

**Table 5:** Physical, emotional or sexual intimate partner violence (IPV) in the preceding year as a possible risk factor for health and healthcare problems described by 2139 slum-dwelling women interviewed at about 6 weeks postpartum

**Dependent variable**	**Did not report**	**Reported**	**OR**	**(95% CI)**	**Adjusted OR**	**(95% CI)**
	**IPV (%)**	**IPV (%)**				
**Illness during pregnancy**						
No illness during pregnancy	1407 (77)	195 (61)	1 ref		1 ref	
Any illness during pregnancy	414 (23)	123 (39)	1.749	(1.32, 2.32)	1.779	(1.32, 2.40)
**Prenatal care received**						
Prenatal care received	1774 (97)	305 (96)	1 ref		1 ref	
No prenatal care received	47 (3)	13 (4)	1.631	(0.86, 3.09)	1.357	(0.68, 2.69)
**Place of delivery**						
Institutional delivery	1644 (90)	280 (88)	1 ref		1 ref	
Home delivery	177 (10)	38 (12)	1.146	(0.77, 1.70)	0.994	(0.66, 1.51)
**Preterm index infant**						
Term	1762 (97)	304 (96)	1 ref		1 ref	
Preterm	50 (3)	11 (3)	1.215	(0.62, 2.39)	1.284	(0.63, 2.63)
Missing	9 (<1)	3 (1)				
Birth weight of index infant *						
Normal	1330 (73)	213 (67)	1 ref		1 ref	
Low birth weight	309 (17)	63 (20)	1.273	(0.94, 1.73)	1.144	(0.83, 1.58)
Missing	182 (10)	42 (13)				
**Family planning**						
Not used modern family planning in last 3 y	1581 (87)	261 (82)	1 ref		1 ref	
Used modern family planning in last 3 y	240 (13)	57 (18)	1.402	(1.01, 1.94)	1.458	(1.02, 2.08)
**Wife beating justifiable in at least one context**						
Not justifiable	1229 (67)	142 (45)	1 ref			
Justifiable	592 (33)	176 (55)	2.36	(1.77, 3.15)	2.260	(1.67, 3.06)
**Total**	**1821 (100)**	**318 (100)**				

Odds ratios (OR) and 95% confidence intervals (CI) from multivariable logistic regression models with random effect for cluster. Models include covariates for socioeconomic quintile, religion, mother’s age and employment, and husband’s alcohol use. Ref: reference category.

* Including a covariate for infant sex did not change the inference.

## Discussion

In interviews with over 2000 women living in Mumbai slums, IPV – physical (12%), emotional (8%) or sexual (2%) – was common during and after pregnancy. Although IPV appears to be less common in urban than in rural settings, new evidence suggests that urban women may be at higher risk after adjustment for socioeconomic status [11]. IPV was more likely to be reported by women in poorer families, Muslim homes, and those whose husbands used alcohol. Although 18% of women who had suffered physical IPV sought clinical care for their injuries, seeking help from organizations outside the family to address IPV itself was rare [14].

Limits to the study included self-report – important given the risk of adverse consequences – and the fact that we used a module within a longer questionnaire administered by researchers who, though trained and a familiar presence within the study areas, were not themselves counselors. They were trained by IPV counselors, were female, and had been interviewing mothers since October 2005. The module on IPV followed less disquieting modules on demography and maternal and newborn care. It was based on modules used in other large surveys, but involved a recall period of up to a year and the questions were relatively closed.

At 15%, reported IPV during maternity accords with other studies from India. Domestic violence during pregnancy (which includes but is not limited to IPV) was described by 18% of respondents in surveys in Uttar Pradesh state in the mid-1990s [29], 21% in a postnatal sample at a Delhi hospital [27], and 28% in pregnant women admitted to hospital in Chandigarh [42]. A large study from Bhopal reported domestic violence in 13% of pregnancies [41], and a multisite study described figures of 26% for physical, 22% for sexual and 63% for psychological violence during pregnancy [26]. The figure of 15% is almost certainly an underestimate: information was incomplete for 17% of the sample, and underreporting is usual in surveys. In some cases, women wanted to share information, but family members were unwilling to ensure privacy in spite of repeated requests by the investigators.

Reports of emotional and sexual IPV were relatively uncommon. We used definitions based on questions comparable with other studies, nested in comparable types of interview. There are two (not mutually exclusive) possibilities for the lower frequencies: that they were underreported or that they were true findings. Achieving participation, confidentiality and disclosure of IPV may have been especially difficult in this study. Mumbai’s slum homes are small, and the practice of confinement to the home in the postpartum period limited the researchers’ likelihood of achieving interviews outside. The second possibility is that rates of emotional and sexual IPV may actually be lower in Mumbai’s poorer communities than in rural settings and some other cities, and during pregnancy and postpartum. To address both these possibilities, we are incorporating service provision for women experiencing IPV within an initiative that integrates multiple activities for women’s and children’s health. The continuous presence of fieldworkers, peer-activists, and counselors in the community allows us to identify IPV prospectively and we hope will contribute to a clearer understanding of its frequency.

In adjusted models, IPV during maternity was more likely in conditions of poverty, Muslim faith, women’s paid employment, and spousal alcohol use. The association with poverty was expected [8,11,12,15,46,54]. As a determinant, faith is more difficult to interpret, but has been noted before [11,27]. Muslim families in Mumbai’s slums are relatively worse-off and it is possible that the increased risk of IPV reflects residual confounding within a matrix of poverty [48,67].

The finding that women who were employed were more likely to have reported IPV is supported by studies across a range of locations in India [10,11,16,43,68]. It has not been replicated in all studies [42]: a small sample in a Kolkata slum suggested that unemployment was a risk factor [12]. Some authors have suggested that women’s work represents a challenge to the patriarchal structure that might provoke spousal violence [11,48,49]. However, employment may be an effect rather than a cause, a means of survival rather than a manifestation of empowerment. A woman may be more likely to seek work if her family is poor, her home environment unstable, and her husband drinks or is having extramarital sex [15,53]. A large study in 18 Indian states suggested that, while working women were at higher risk of IPV, women engaged in unskilled labour were most at risk [7]; and a study from Mysore suggested that, although women with jobs were more likely to suffer IPV than women without jobs, those with skilled occupations were at lower risk [69]. Chibber and colleagues suggest that women who contribute to household income are at greater risk than non-contributors, but women who are solely responsible for household income are at lower risk [49]. Perhaps stability and reliability within relationships are important. When women have more resources in terms of land and assets, IPV seems to be less common [16,54]. A study involving 744 married women in Bangalore slums suggested that the risk of IPV increased if unemployed women became employed, or if their husbands lost their jobs [70]. It also suggested that violence was more common in love marriages, women who worked before and after marriage, and participants in social and vocational groups. Because of social structures in India, it is possible that love marriages are more likely to be accompanied by financial adversity and involve emotional strain.

Spousal alcohol use is a determinant of IPV [10-13,15,41,42,51,71]. We did not see increased odds of IPV in older or less educated women, or in those who had married younger, which have been described [11,13,55,56]. We speculated that the risk of IPV might be higher if there were substantial differences in education or age between husband and wife [11,12,50,58], but we saw no evidence for this in subsidiary analyses.

Although spousal drunkenness, suspected infidelity, unwillingness to have sex, and dowry were not now seen as justifiable triggers, one-third of women felt that violence was a justifiable response to what might be described as a failure to live up to the role of wife and mother within her husband’s family. This finding echoes those of Jejeebhoy in rural Uttar Pradesh and Tamil Nadu [54]. However, a recent analysis of the NFHS-3 found that women who justified partner violence were less likely to suffer it. The authors suggest that this might reflect submissive avoidance [11]. We found the opposite: that women who reported IPV were more than twice as likely to say that there were situations in which it was justifiable. Whether this represented self-protective rationalization or was part of the causal matrix is unclear. There is also some evidence that justification is associated with less likelihood of care-seeking for illness. For example an analysis of NFHS-3 data from Uttar Pradesh suggested that women who accepted any justification of violence were less likely to seek care for sexually transmitted infections [38].

IPV during pregnancy has implications for the health and wellbeing of mothers and fetuses, and with less likelihood of prenatal care and care for intercurrent problems [26,34]. Studies have found that physical IPV in pregnancy increases the likelihood of miscarriage or low birth weight [31,72-76]. Again, we are unsure about the cause structure. One could sketch a pathway from IPV to compromised fetal growth and miscarriage. Equally, one could propose that the association is residually confounded by the socioeconomic and cultural milieu, so that poor outcomes are corollaries of IPV but not caused by it. For example, miscarriage may be the result of IPV, but may also add to family stress and make subsequent violence more likely. IPV during pregnancy may lead to illness (early labour and vaginal bleeding were mentioned by respondents), but concerns about IPV might also increase the likelihood that a woman will report illness when asked about it in an interview. Ackerson and Subramanian found a strong association between IPV and increased child mortality rates, and suggested that violence impaired women’s ability to take care of their children and caused psychological stress in the children themselves [32]. Some of our respondents reported difficulty in taking care of themselves during pregnancy and difficulty in caring for their babies.

Unwanted pregnancies and the number of living children have been associated with IPV in several studies [32,77-80]. The increased likelihood of family planning in women who reported IPV is counterintuitive, since it is a lack of control over women’s reproductive health choices that has been discussed previously [80,81]. It is just possible that women are making choices to limit conception in a stressful situation, but we emphasize that we have no evidence for this intriguing speculation.

## Conclusions

Intimate partner violence against women during maternity was unacceptably common in Mumbai’s slums. One in seven women suffered violence during or shortly after pregnancy. IPV begins in a culture that condones it – indeed, justifies it - and is abetted by poverty and alcohol use. The elements of the violent milieu are mutually reinforcing and need to be taken into account collectively in responding to both individual cases and framing public health initiatives.

## Competing interests

The authors declare that they have no competing interest.

## Authors’ contributions

All authors contributed to the design of the study. SD supervised data collection, carried out the main analysis and wrote the first draft. UB supervised field activities and data collection. NSM was the project director. GA was technical adviser to the project. WJ and SP had overall responsibility for SNEHA programmes. DO helped with the analysis and edited drafts of the manuscript. All authors read and approved the final manuscript.

## Pre-publication history

The pre-publication history for this paper can be accessed here:

http://www.biomedcentral.com/1471-2458/13/817/prepub
